# The First Tracheotomy with the Aid of the Galvano-Cautery, Etc.

**Published:** 1873-05

**Authors:** 


### THE FIRST TRACHEOTOMY WITH THE AID OF THE GALVANO-CAUTERY, IN GERMANY, FOR MORBID GROWTH CLOSING THE GLOTTIS FROM BELOW.

Verneuil, in France, was the first one who performed this operation in this manner, and Voltolini, in Germany, the second.

The subject was a wealthy farmer. The disease a berry-like growth within the larynx, obstructing the glottis from below, the result of chronic laryngitis, caused by excessive exposure in the late Franco-Germanic war. Aphonia, emphysema of both lungs, high-graded dyspnœa, slight cough and expectoration, and acceleration of the pulse, were the prominent symptoms. Appetite good, but the threatening danger of suffocation demanded a removal of the morbid growth.

Repeated efforts to remove it through the mouth did not succeed, on account of unfavorable local conditions, backward inclination of the glottis, etc.; and laryngo-tracheotomy, with removal of the morbid growth through the tracheal opening, was agreed upon by Dr. Voltolini and Dr. Reichel, and, at the proposition of the latter, with the aid of the galvano-cautery.

A small platina scalpel was used as galvano-cautery. It penetrated all parts without the slightest effort or resistance, almost without any hemorrhage; but after the patient had, at the commencement of the operation, been only with difficulty rescued from the unfavorable effects of chloroform, the sudden entrance of air through the canula into the tracheal opening produced again an alarming collapse; from which the patient could only be aroused with the very greatest difficulty. Under these circumstances, it was deemed imprudent to attempt the removal of the morbid growth at once. On laryngeal examination on the fourteenth day after the operation, the same could not be discovered, it was almost entirely gone, and the patient could breathe freely with the tracheal opening closed by pressure, and blow air through the nose.

Professor Voltolini's acknowledged skill in laryngoscopic examinations, and the excessive dyspnœa which required the operation, exclude the idea of a delusion in relation to the existence of a new growth within the larynx; and therefore only the supposition that the growth situated immediately below the vocal chords, which extend nearly to the lower edge of the thyroid cartillage was touched by the heated cautery when it penetrated the lower edge of the ligamentum crico-thyrioideum and was thus destroyed, and afterward vanished by suppuration.

Voltolini claims the entire painlessness and the very trifling hemorrhage as the advantages of the galvano-caustic method of performing tracheotomy. Verneuil's observations in relation to these points coincide with those of Voltolini, and while the two operations have not brought anything to light to forbid its execution, it will have to be performed many more times before its real surgical value can be properly defined.—*Berliner Klinische Wochenschrift, No.* 41, 1872. R.

Dr. Frank A. Ramsey.—We learn that this eminent medical gentleman will spend the season at Montvale Springs, East Tennessee, which will doubtless prove another source of attraction to that delightful and health-restoring resort.

